# Supplementary material for: Development of smart anti-glycan reagents using immunized lampreys
Source: Commun Biol. 2020 Feb 28;3:91. doi: 10.1038/s42003-020-0819-2 (PMC7048801; doi:10.1038/s42003-020-0819-2)
Supplement: Supplementary file 1 — Supplementary Information [file 42003_2020_819_MOESM1_ESM.pdf]

a.

	A. Naïve	B. CHO Pro.5	C. Lec8	D. Lec8GT	E. Lec8GTFT	F. Pig Lung	G. Tn4	H. Human Milk	I. Type O RBC	J. Type AB RBC	K. SIV Particles
A. Naïve	1.0000										
B. CHO Pro.5	0.0287	1.0000									
C. Lec8	0.0215	**0.4928**	1.0000								
D. Lec8GT	0.0042	0.3969	**0.4506**	1.0000							
E. Lec8GTFT	0.0099	0.3186	**0.5251**	**0.6768**	1.0000						
F. Pig Lung	0.0070	**0.4754**	0.2055	0.2088	0.0698	1.0000					
G. Tn4	0.0005	-0.0030	0.3670	0.0865	0.0491	0.0822	1.0000				
H. Human Milk	0.0279	0.3653	0.3822	0.1755	0.2027	0.3169	-0.0145	1.0000			
I. Type O Erythrocytes	0.0118	**0.4519**	0.0828	0.1139	0.0051	**0.6939**	0.0045	0.1383	1.0000		
J. Type AB Erythrocytes	0.0036	0.3855	0.2219	0.2377	0.0722	**0.6757**	0.1094	0.2994	**0.6735**	1.0000	
K. SIV Particles	-0.0043	0.0377	0.3184	0.0936	0.0479	0.0800	**0.6852**	0.1055	0.0254	0.0993	1.0000

b.

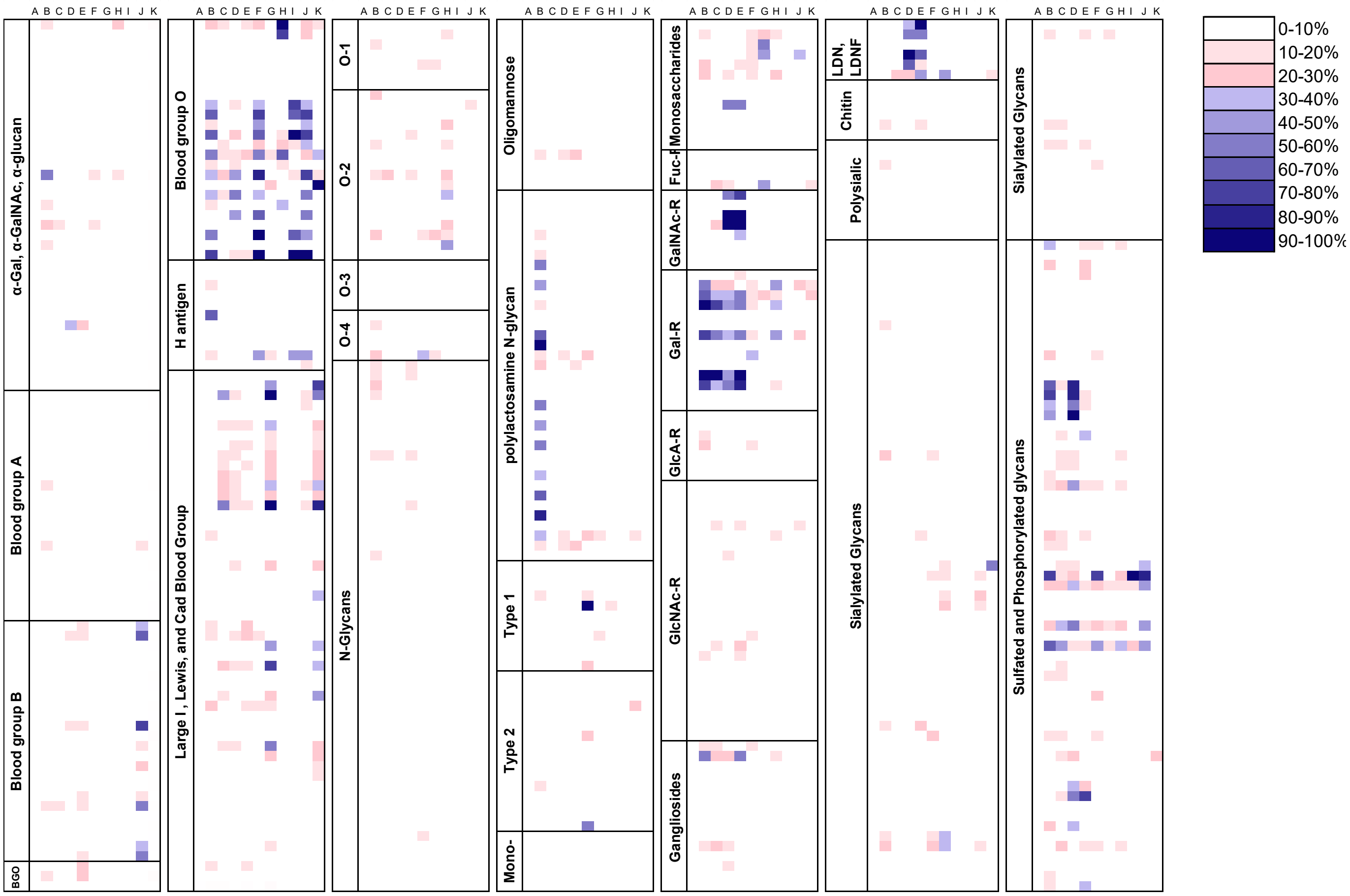

Supplementary Figure 1. Ranked binding profiles of the libraries on the CFG array, as Pearson correlation (a) and heat maps (b) by percent rank. Each vertical line of the heatmap represents individual lamprey serum immunized with: A. Naïve, B. CHO - Pro.5, C. CHO - Lec8, D. CHO - Lec8GT, E. CHO - Lec8GTFT, F. Pig Lung, G. Tn4 B cells, H. Human milk, I. Type O erythrocytes, J. Type AB erythrocytes, K. SIV particles.

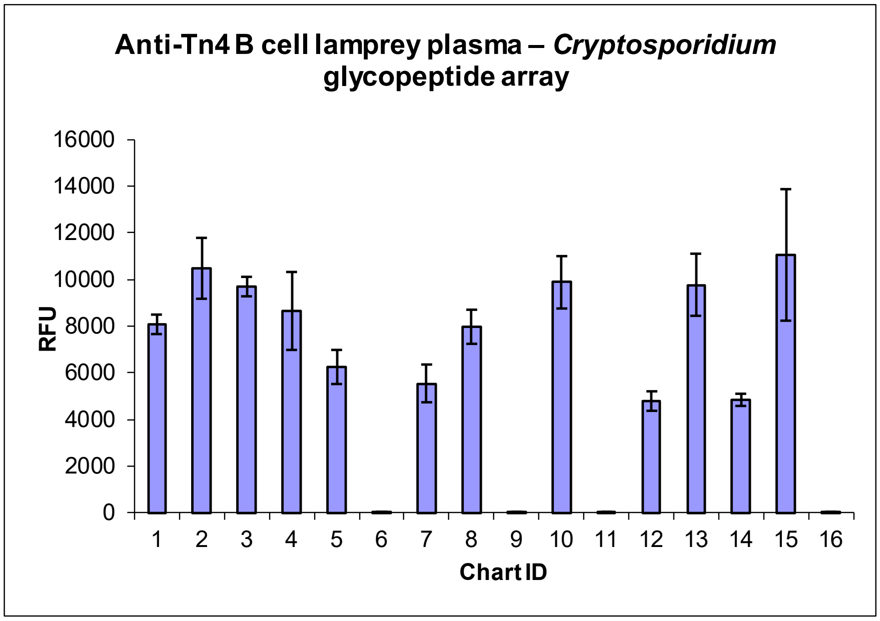

ID	Sequence	RFU	STDEV	CV%
1	H-ETS*EAAAT*VDLFAFT*LDGGK-NH2	8086	406	5
2	H-ETSEAAAT*VDLFAFT*LDGGK-NH2	10496	1307	12
3	H-ETS*EAAATVDLFAFT*LDGGK-NH2	9677	428	4
4	H-ETSEAAATVDLFAFT*LDGGK-NH2	8650	1648	19
5	H-ETS*EAAAT*VDLFAFTLDGGK-NH2	6255	726	12
6	H-ETSEAAAT*VDLFAFTLDGGK-NH2	16	3	17
7	H-ETS*EAAATVDLFAFTLDGGK-NH2	5528	808	15
8	H-ETSEAAATVDLFAFTLDGGK-NH2	7987	735	9
9	H-ETT*EAAAS*VDLFAFS*LDGGK-NH2	19	9	48
10	H-ETTEAAASVDLFAFSLDGGK-NH2	9883	1110	11
11	H-DVPVEGSS*(7)TSTVAPANK-NH2	15	4	27
12	H-DVPVEGSS(7)TSTVAPANK-NH2	4792	433	9
13	H-DVPVEGSS*(16)TSTVAPANK-NH2	9761	1334	14
14	H-DVPVEGSS(16)TSTVAPANK-NH2	4820	253	5
15	H-DVPVEGSS*(23)TSTVAPANK-NH2	11061	2813	25
16	H-DVPVEGSS(23)TSTVAPANK-NH2	18	3	16

Supplementary Figure 2. Serum from lamprey immunized with the Tn4 B cells was screened on the Cryptosporidium glycopeptide array, suggests anti-Tn VLRBs are present in the sample, buy may require multiple repeated Tn epitopes for binding.

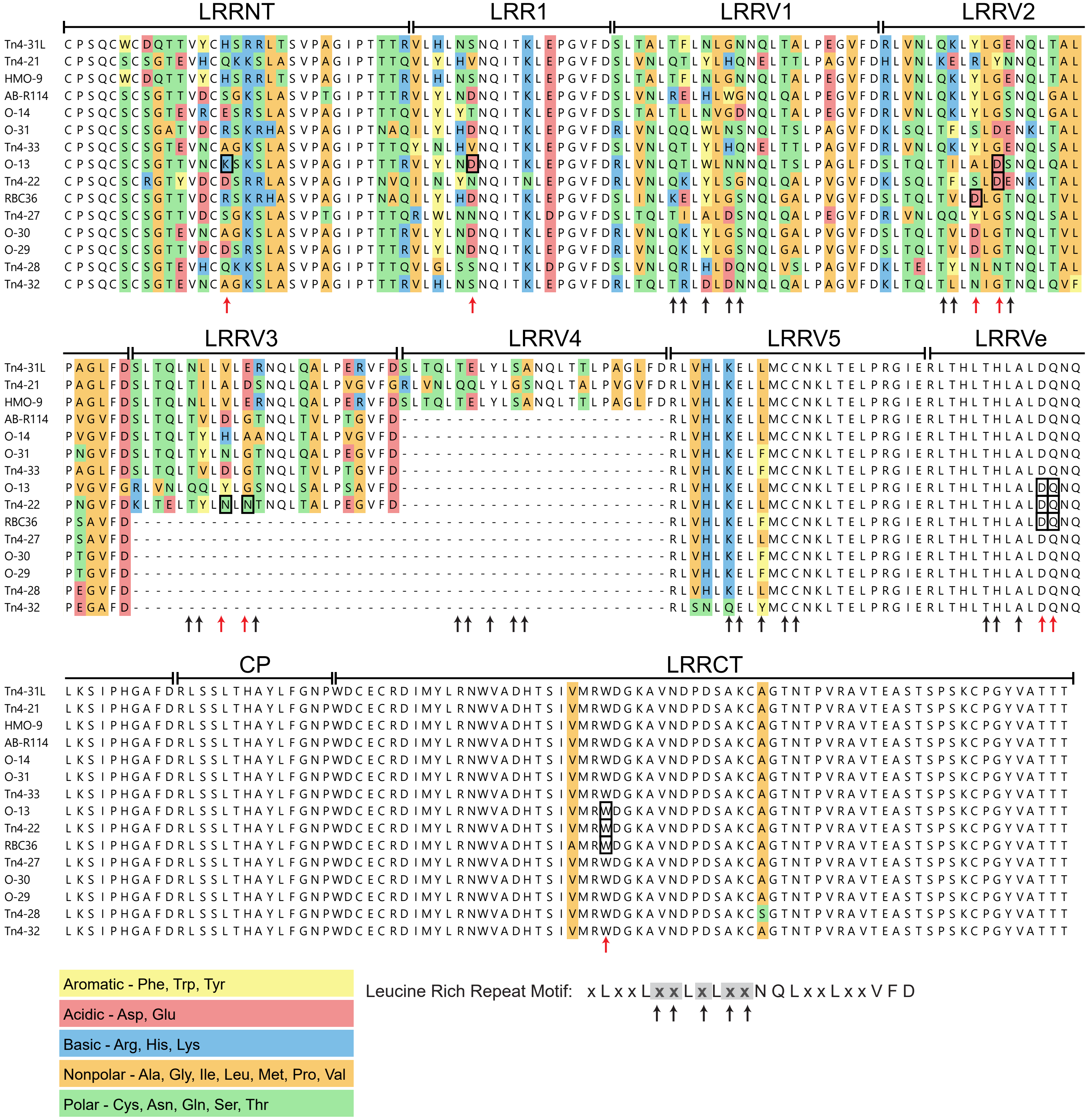

Supplementary Figure 3. Amino acid sequence alignment of the H-antigen VLRB monoclonals using ClustalW. Arrows are pointing to the amino acid residues that are structurally facing the concave surface of the binding pocket, and could potentially come into contact with the antigen. Red arrows are pointing to residues known to interact with sugar residues from previously published crystallography data, and these residues are highlighted with a black box.

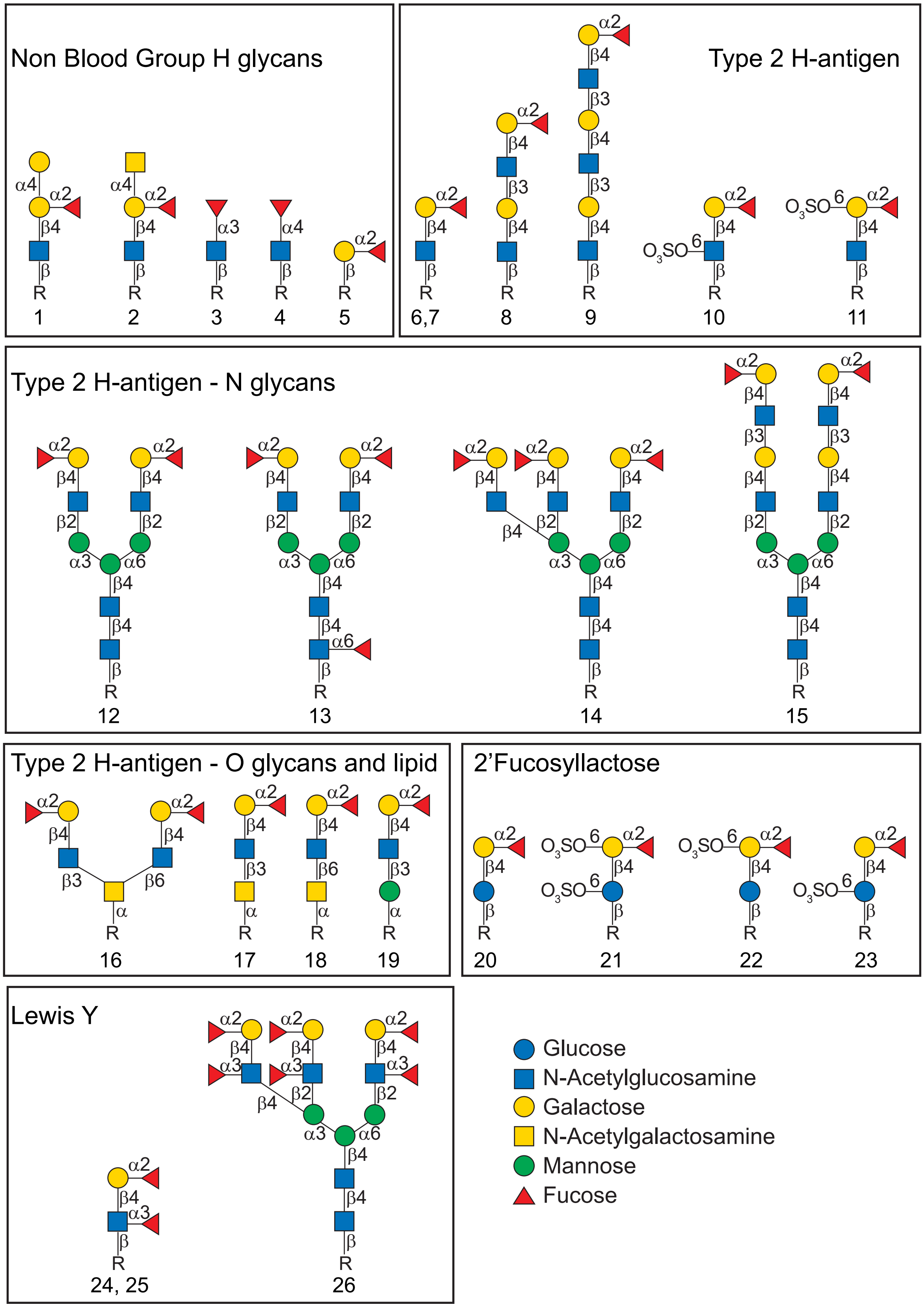

Supplementary Figure 4. Glycan structures of the type 2 H-antigen used in **Figure 5**. Glycans with two numbers listed (6,7 and 24,25) were printed on the arrays with different linkers.

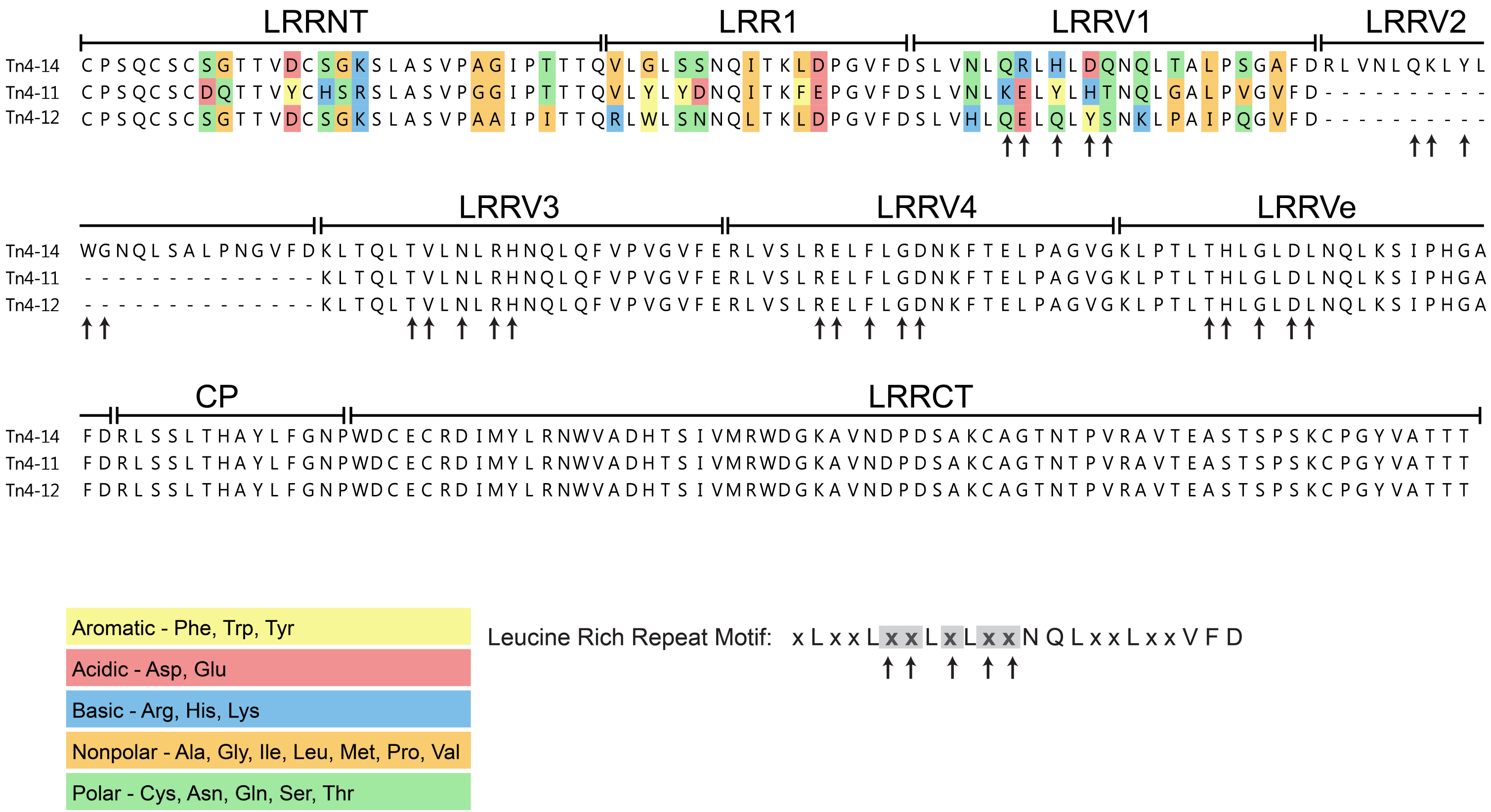

Percent Similarity in upper triangle
Pearson r in lower triangle

	Tn4-14	Tn4-11	Tn4-12
Tn4-14	***	82.3	83.9
Tn4-11	0.398	***	89.9
Tn4-12	0.627	0.598	***

Supplementary Figure 5. Amino acid sequence alignment of the sialic acid specific VLRB monoclonals using ClustalW. Arrows are pointing to the amino acid residues that are structurally facing the concave surface of the binding p ocket, and could potentially come into contact with the antigen. Sequence ali gnments and distances were calculated using ClustalW and MegAlign. Pearson r correlation was calculated based on the array profiling data illustrated in **Figure 6**.

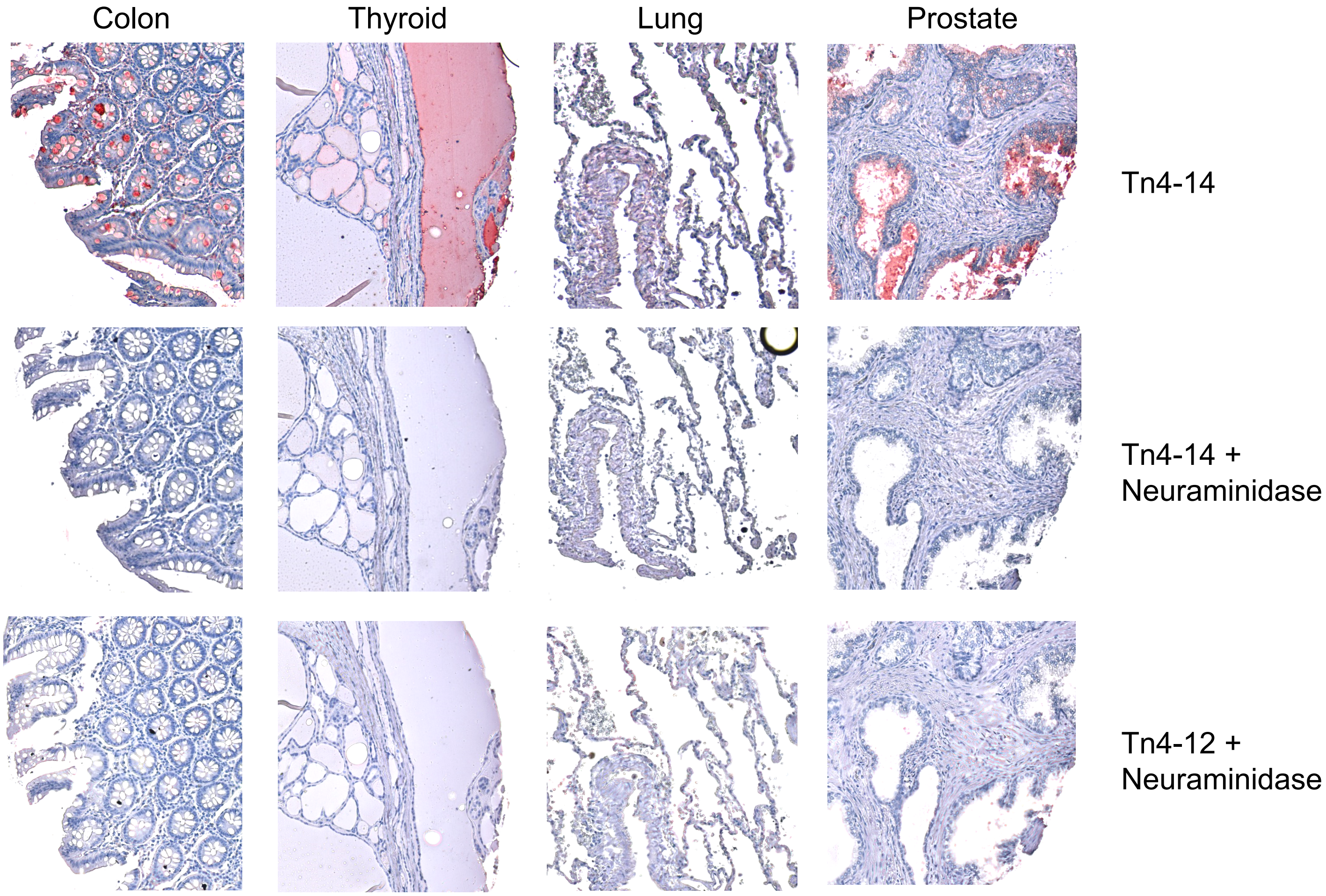

Supplementary Figure 6. Human tissue arrays were treated with Neuraminidase and stained with Tn4-14 and Tn4-12 as previously described. The Neuraminidase treatment clearly abolished all staining with the antibodies, demonstrating the specificity of the binding to sialylated glycans.
