# Supplementary material for: Development of smart anti-glycan reagents using immunized lampreys
Source: Commun Biol. 2020 Feb 28;3:91. doi: 10.1038/s42003-020-0819-2 (PMC7048801; doi:10.1038/s42003-020-0819-2)
Supplement: Supplementary file 2 — Description of Additional Supplementary Files [file 42003_2020_819_MOESM2_ESM.pdf]

Description of additional supplementary files

Supplementary data 1 contains all of the lamprey plasma and mouse serum run on the CFG array. Each individual tab is labeled by the antibody type and immunogen. Note: *a/b* = *α/β* linkage.

Supplementary data 2 contains all of the data collected from the yeast bound to the array. This includes the libraries as well as the RBC36 clone. Each tab is labeled by the sample. Note: *a/b* = *α/β* linkage.

Supplementary data 3 contains all of the data from each monoclonal antibody on the CFG array and NCFG Sialyl Derivative array. Each tab is labeled by sample and array type. Note: *a/b* = *α/β* linkage.
