# Supplementary material for: Development of smart anti-glycan reagents using immunized lampreys
Source: Commun Biol. 2020 Feb 28;3:91. doi: 10.1038/s42003-020-0819-2 (PMC7048801; doi:10.1038/s42003-020-0819-2)
Supplement: Supplementary file 6 — Reporting Summary [file 42003_2020_819_MOESM6_ESM.pdf]

nature research

Corresponding author(s): Richard Cummings

Last updated by author(s): Nov 22, 2019

Reporting Summary

Nature Research wishes to improve the reproducibility of the work that we publish. This form provides structure for consistency and transparency in reporting. For further information on Nature Research policies, see Authors & Referees and the Editorial Policy Checklist.

Statistics

For all statistical analyses, confirm that the following items are present in the figure legend, table legend, main text, or Methods section.

n/a	Confirmed	
☒	☐	The exact sample size (n) for each experimental group/condition, given as a discrete number and unit of measurement
☒	☐	A statement on whether measurements were taken from distinct samples or whether the same sample was measured repeatedly
☒	☐	The statistical test(s) used AND whether they are one- or two-sided<br>*Only common tests should be described solely by name; describe more complex techniques in the Methods section.*
☒	☐	A description of all covariates tested
☒	☐	A description of any assumptions or corrections, such as tests of normality and adjustment for multiple comparisons
☐	☒	A full description of the statistical parameters including central tendency (e.g. means) or other basic estimates (e.g. regression coefficient) AND variation (e.g. standard deviation) or associated estimates of uncertainty (e.g. confidence intervals)
☒	☐	For null hypothesis testing, the test statistic (e.g. F, t, r) with confidence intervals, effect sizes, degrees of freedom and P value noted<br>*Give P values as exact values whenever suitable.*
☒	☐	For Bayesian analysis, information on the choice of priors and Markov chain Monte Carlo settings
☒	☐	For hierarchical and complex designs, identification of the appropriate level for tests and full reporting of outcomes
☒	☐	Estimates of effect sizes (e.g. Cohen's d, Pearson's r), indicating how they were calculated

Our web collection on statistics for biologists contains articles on many of the points above.

Software and code

Policy information about availability of computer code

Data collection	no software was used
Data analysis	no software was used

For manuscripts utilizing custom algorithms or software that are central to the research but not yet described in published literature, software must be made available to editors/reviewers. We strongly encourage code deposition in a community repository (e.g. GitHub). See the Nature Research guidelines for submitting code & software for further information.

Data

Policy information about availability of data

All manuscripts must include a data availability statement. This statement should provide the following information, where applicable:

- Accession codes, unique identifiers, or web links for publicly available datasets
- A list of figures that have associated raw data
- A description of any restrictions on data availability

All raw microarray data from all figures is provided in the supplementary files

Field-specific reporting

Please select the one below that is the best fit for your research. If you are not sure, read the appropriate sections before making your selection.

☒ Life sciences ☐ Behavioural & social sciences ☐ Ecological, evolutionary & environmental sciences

For a reference copy of the document with all sections, see nature.com/documents/nr-reporting-summary-flat.pdf

Life sciences study design

All studies must disclose on these points even when the disclosure is negative.

Sample size	Three animals were immunized with antigen, and screened for the highest antigen specific titers as described in the methods. The animal with the highest measurable titer was chosen for glycan microarray screening and to generate YSD libraries.
Data exclusions	No data was excluded
Replication	Replication of immunizations is not possible, as these are wild type animals and we cannot control for prior exposure to common antigens. The glycan microarrays contain replicates of 6 of each glycan to ensure the technical robustness of the assay.
Randomization	This is not relevant to our study
Blinding	This is not relevant to our study

Reporting for specific materials, systems and methods

We require information from authors about some types of materials, experimental systems and methods used in many studies. Here, indicate whether each material, system or method listed is relevant to your study. If you are not sure if a list item applies to your research, read the appropriate section before selecting a response.

Materials & experimental systems

n/a	Involved in the study	
☐	☒	Antibodies
☐	☒	Eukaryotic cell lines
☒	☐	Palaeontology
☐	☒	Animals and other organisms
☒	☐	Human research participants
☒	☐	Clinical data

Methods

n/a	Involved in the study	
☒	☐	ChIP-seq
☒	☐	Flow cytometry
☒	☐	MRI-based neuroimaging

Antibodies

Antibodies used	Anti-VLRB mAb 4C4 produced in Max Cooper's laboratory, goat anti-mouse IgG, Life technologies Cat# A11001, goat anti-mouse IgM, Invitrogen Cat# A21046, goat anti-rabbit IgG (H+L),Invitrogen A11034, Peroxidase-conjugated AffiniPure Goat Anti-Rabbit IgG (H+L), Jackson Immunoresearch cat# 111-035-144, Anti-Myc-488, EMD Millipore, Cat# 16-224
Validation	Secondary antibody controls are printed on the glycan microarray to validate specificity of fluorescent. Secondary only control screens were performed for IHC.

Eukaryotic cell lines

Policy information about cell lines

Cell line source(s)	FreeStyle 293-F, Thermofisher
Authentication	none of the cell lines were authenticated
Mycoplasma contamination	cell lines were not tested for mycoplasma
Commonly misidentified lines (See ICLAC register)	NA

Animals and other organisms

Policy information about studies involving animals; ARRIVE guidelines recommended for reporting animal research

Laboratory animals	Six-to-eight week old BALB/cAnNHsd mice were obtained from Harlan (Indianapolis, IN) , as stated in the methods
Wild animals	Petromyzon marinus larvae (8-15 cm in length, approximately 2-4 years in age) were collected from tributaries to Lake Michigan (Lamprey Services, Ludington, MI) and housed in sand-lined aquarium tanks at 20°C in the animal facility at Emory University as stated in the methods
Field-collected samples	N/A

Ethics oversight | IACUC approvals from Emory University were obtained for work with lampreys

Note that full information on the approval of the study protocol must also be provided in the manuscript.
